# Artificial Intelligence Diagnosing of Oral Lichen Planus: A Comparative Study

**DOI:** 10.3390/bioengineering11111159

**Published:** 2024-11-18

**Authors:** Sensen Yu, Wansu Sun, Dawei Mi, Siyu Jin, Xing Wu, Baojian Xin, Hengguo Zhang, Yuanyin Wang, Xiaoyu Sun, Xin He

**Affiliations:** 1 Key Laboratory of Oral Diseases Research of Anhui Province, College & Hospital of Stomatology, Anhui Medical University, Hefei 230032, China; yu0202133@163.com (S.Y.); daweimi2016@163.com (D.M.); 15872709832@163.com (S.J.); xwu0106@163.com (X.W.); 2020500057@ahmu.edu.cn (B.X.); 2 Department of Stomatology, The First Affiliated Hospital of Anhui Medical University, Hefei 230032, China; sunwansu@ahmu.edu.cn; 3 Department of Stomatology, Suzhou Hospital of Anhui Medical University, Suzhou 234099, China

**Keywords:** oral lichen planus, artificial intelligence, ChatGPT, auxiliary diagnosis, image recognition

## Abstract

Early diagnosis of oral lichen planus (OLP) is challenging, which traditionally is dependent on clinical experience and subjective interpretation. Artificial intelligence (AI) technology has been widely applied in objective and rapid diagnoses. In this study, we aim to investigate the potential of AI diagnosis in OLP and evaluate its effectiveness in improving diagnostic accuracy and accelerating clinical decision making. A total of 128 confirmed OLP patients were included, and lesion images from various anatomical sites were collected. The diagnosis was performed using AI platforms, including ChatGPT-4O, ChatGPT (Diagram-Date extension), and Claude Opus, for AI directly identification and AI pre-training identification. After OLP feature training, the diagnostic accuracy of the AI platforms significantly improved, with the overall recognition rates of ChatGPT-4O, ChatGPT (Diagram-Date extension), and Claude Opus increasing from 59%, 68%, and 15% to 77%, 80%, and 50%, respectively. Additionally, the pre-training recognition rates for buccal mucosa reached 94%, 93%, and 56%, respectively. However, the AI platforms performed less effectively when recognizing lesions in less common sites and complex cases; for instance, the pre-training recognition rates for the gums were only 60%, 60%, and 20%, demonstrating significant limitations. The study highlights the strengths and limitations of different AI technologies and provides a reference for future AI applications in oral medicine.

## 1. Introduction

Oral lichen planus (OLP) is a prevalent chronic inflammatory condition of the oral mucosa, predominantly affecting middle-aged and older females [1]. Epidemiological statistics indicate that the incidence of OLP in the global adult population ranges from 0.5% to 2% [2]. This relatively high incidence underscores the public health significance of the disease, particularly among specific populations such as the elderly and female patients, where its impact is notably pronounced. OLP can considerably impair quality of life and lead to various complications, including oral pain, eating discomfort, and difficulties in social interactions, all of which directly affect psychological well-being and daily activities [3].

Clinically, OLP typically presents with lesions that exhibit bilateral symmetry, characterized by reticular, annular, or plaque-like white patches, occasionally accompanied by erythema and erosions. Histopathological examination reveals distinct features, such as band-like lymphocytic infiltration, intraepithelial capillary dilation, and liquefactive degeneration of the basal cells. These pathological characteristics complicate the diagnosis of OLP [4]. Based on lesion type, OLP can be categorized into several subtypes, including reticular, plaque, atrophic, erosive, and vesicular types. The significant morphological, structural, and severity differences among these subtypes increase the challenges clinicians face in visual diagnosis. Prolonged cases often require extensive follow-up, further straining clinicians’ time and resources. Notably, OLP is classified as a potentially malignant disorder by the World Health Organization (WHO), with a malignant transformation rate of up to 2% [5]. Therefore, early identification and ongoing surveillance are crucial for improving patient outcomes [6]. Although oral medications and systemic corticosteroid therapy can play a role in treatment, early intervention remains the most effective therapeutic strategy to prevent disease progression.

Currently, the diagnosis of OLP primarily relies on clinical examinations and histopathological assessments. However, practical clinical settings reveal several challenges to establishing a definitive diagnosis. First, the manifestations of OLP lack specificity and can easily be mistaken for other oral mucosal diseases, such as oral leukoplakia and lupus erythematosus. The similar characteristics of these conditions often lead to misdiagnosis by some clinicians. Second, the invasive nature and limitations of tissue biopsy restrict its routine application, especially for patients with severe disease and multiple sites of involvement. Even when pathological sampling is conducted, accurate interpretation of histopathological slides requires extensive expertise, and subjective differences among pathologists can influence final diagnostic outcomes [7]. Thus, early diagnosis of OLP presents a significant challenge for both patients and physicians, necessitating dynamic assessments of disease changes through continuous and frequent follow-ups [8,9].

With the rapid advancement of artificial intelligence (AI) technology in recent years, the application of AI platforms for medical disease diagnosis has garnered considerable interest [10,11]. The core principle of AI in case image recognition is based on deep learning algorithms, which simulate human neural networks to autonomously learn and extract disease features from extensive medical image datasets [12]. This process establishes an end-to-end mapping from input images to diagnostic outcomes [13]. In contrast to traditional computer-aided diagnostic systems, AI platforms can autonomously identify more precise and nuanced hierarchical feature representations, showcasing their robust capabilities in data processing and analysis [14]. Recently, AI has demonstrated diagnostic performance comparable to that of clinical specialists across various medical fields, including dermatology, fundus lesions, respiratory diseases, dental caries, and orthopedic surgery [15,16,17,18,19,20,21]. This advancement will undoubtedly facilitate the transformation of medical practice towards greater intelligence, thereby enhancing the quality of medical services provided to patients [22]. AI diagnosing OLP is anticipated to address the limitations of traditional visual assessments and subjective judgments, thereby facilitating objective diagnosis and precise lesion classification [23].

In this study, we aim to provide relevant evidence to support the development and application of OLP-related AI diagnosis. We validated that the utilization of AI for the diagnosis and recognition of OLP is expected to significantly enhance both efficiency and accuracy in clinical practice. Through systematic data analysis and advanced pattern recognition, AI is anticipated to play a pivotal role in the early detection and management of OLP. The effective integration of AI technology into clinical workflows can provide real-time auxiliary diagnostic information, thereby assisting physicians in making more informed and precise decisions when addressing complex cases. We anticipate that future AI diagnostic systems will not only improve the recognition rate of OLP but also integrate additional clinical information for a multidimensional comprehensive assessment.

## 2. Methods

### 2.1. Case Collection

During the sample collection phase of this study, clinical images were gathered from 128 patients diagnosed with oral lichen planus (OLP). To ensure the study’s rigor and reliability, three experienced oral mucosal disease specialists independently reviewed the collected images. By integrating clinical expertise with relevant diagnostic guidelines and the latest International Classification of Diseases (ICD-11), the images from the collected clinical cases were meticulously evaluated. Only those patients whose images meet the diagnostic criteria and pass expert assessment were included in the final analysis, thereby ensuring the validity and accuracy of the study. The exclusion criteria included patients with concurrent oral mucosal diseases, those who had received systemic treatment or related mucosal surgeries, as well as cases of lichen-like lesions caused by other factors or occurrences of the disease in extremely rare locations.

### 2.2. Image Acquisition and Preprocessing

Professional oral mucosal disease specialists recorded images of the lesions in patients according to standardized photographic parameters. One to three representative images of the lesions were selected for each patient, along with documentation of the lesion sites. After image collection, the original images were standardized using Photoshop software, including uniform resolution, white balance correction, and removal of irrelevant backgrounds. Additionally, to protect patient privacy, areas involving facial features of the patients were blurred.

### 2.3. AI Platform Selection

This study selected several mainstream AI platforms, including ChatGPT-4O, the Chat-Diagrams plugin, and Claude Opus, for a comparative analysis of their diagnostic performance. Following the predefined experimental methods, three AI models were established and calibrated for subsequent performance evaluation.

### 2.4. AI Diagnostic Process

The experiment was conducted in two groups (pre-training and non-pre-training). In the non-pre-training group, each AI platform was tested directly for its diagnostic capabilities on the collected images. In the pre-training group, which served as a comparative identification test, we performed standardized pre-training of the AI platforms prior to initiating the diagnosis. Specifically, we provided a textual description of the clinical presentations and imaging characteristics of oral lichen planus (OLP) at various oral sites. To avoid interference between recognition results, each identification session was conducted in completely independent scenarios each time. Subsequently, the diagnostic performance of each platform was statistically analyzed and evaluated.

### 2.5. Diagnostic Performance Evaluation

The recognition accuracy of the three AI platforms was statistically analyzed and compared across different oral sites and batches to evaluate their diagnostic performance

### 2.6. Statistical Analysis

Data analysis was performed using SPSS 27.0 software. We labeled the recognition results of each case image as either correct or incorrect. Using the bootstrapping statistical method, we performed resampling with replacement on the entire case dataset. This process was repeated numerous times to estimate the mean and the corresponding 95% confidence interval, and inter-group comparisons were conducted using one-way ANOVA. A *p*-value of less than 0.05 was considered statistically significant [18].

## 3. Result

**Figure 1:**
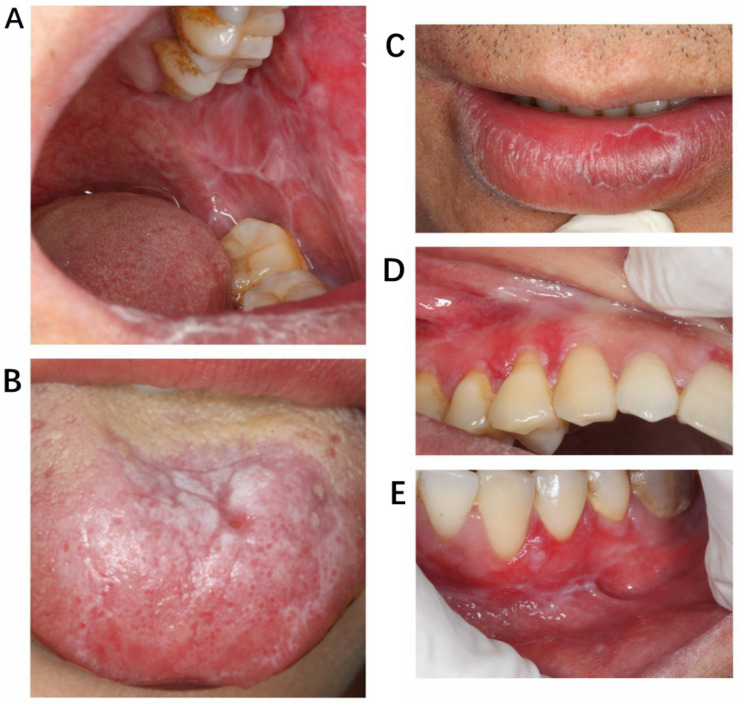
The case images shown in the figure are selected from different patients and various affected areas, including the main sites of OLP involvement. (**A**) Buccal mucosa, (**B**) tongue, (**C**) vermilion of the lips, (**D**) gingiva, (**E**) vestibular groove.

To more accurately simulate a clinical diagnostic environment, we followed relevant guidelines to determine lesion locations and imaging standards for the study [24]. This approach ensures that the study emphasizes commonly affected clinical sites, with sample sizes for each location roughly aligning with their incidence rates (Figure 1). Additionally, we compiled the basic patient information and presented it in a table, as shown in Table 1.

According to Table 2, following the learning of diagnostic characteristics for OLP at various locations, the diagnostic accuracy of the three AI platforms exceeded that of the non-pre-training group. Among them, the trained Chat-Diagrams performed the best, achieving an accuracy of 80%, surpassing ChatGPT-4O (77%) and Claude Opus (50%).

**Figure 2:**
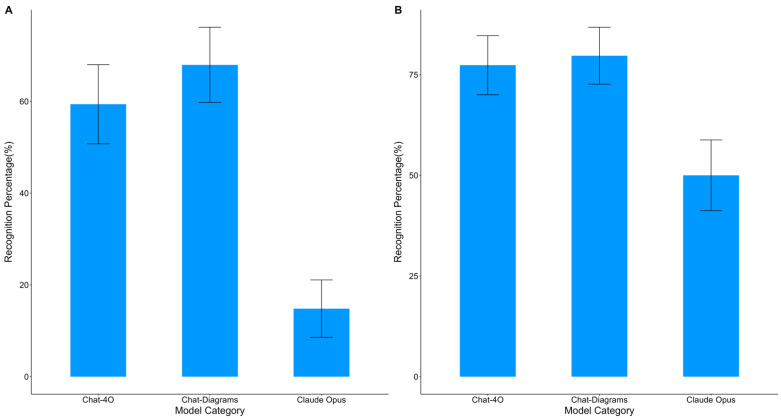
(**A**) The recognition accuracy of various platforms with non-pre-training group. (**B**) The recognition accuracy of various platforms with pre-training group.

However, when we conducted simple image recognition directly on the AI platforms, their performance was somewhat lacking, yielding results of 59%, 68%, and 15%, respectively (Figure 2). According to Table 3, both before and after training, the three platforms displayed notable statistical differences in recognition performance (*p* < 0.05), suggesting some variation in recognition efficacy. It is noteworthy that there was no significant statistical difference in recognition performance between the trained ChatGPT-4O and Chat-Diagrams (*p* > 0.05)

**Figure 3:**
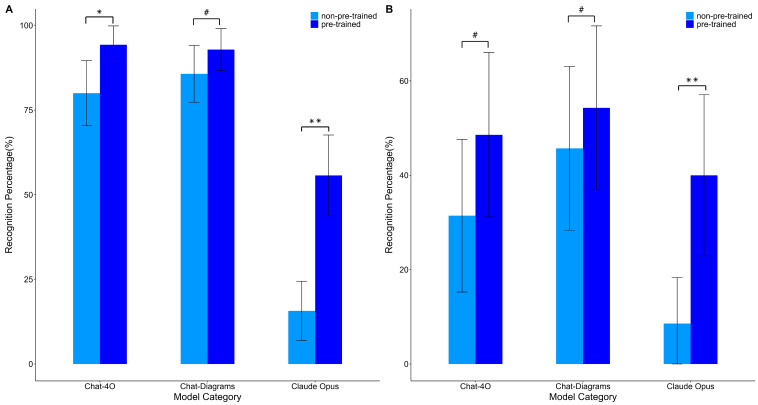
(**A**) The recognition performance of various platforms on the buccal mucosa before and after training. (**B**) The recognition performance of various platforms on the tongue body before and after training. * *p* < 0.05; ** *p* < 0.01, ^#^ *p* > 0.05 by one-way ANOVA.

AI exhibits significant variations in diagnostic performance across different oral sites. According to the data in Table 2, the AI system performs optimally in commonly affected areas, such as the buccal mucosa, significantly outperforming its effectiveness in the tongue, gums, and other sites (Figure 3). This is likely due to the more distinct image features of lesions in the buccal mucosa, while lesions in other areas are more complex and their clinical manifestations may be confused with other diseases, increasing the difficulty for the AI platforms in recognition. In the diagnosis of buccal mucosa lesions after training, the diagnostic accuracy rates of ChatGPT-4O, Chat-Diagrams, and Claude Opus were 94.0%, 93.0%, and 56%, respectively, with ChatGPT-4O showing the most remarkable performance. When we focus on the recognition performance in less common affected areas, the results from the three platforms were somewhat disappointing. For example, in the recognition of lesions in the gums, the pre-training group performance of the three platforms was 30%, 10%, and 10%, respectively. After training, both ChatGPT-4O and Chat-Diagrams improved their accuracy rates to 60%. The training effect for Claude Opus appears to be less pronounced, possibly due to the limited sample size. In recognizing lesions on the vermilion border, both ChatGPT-4O and Chat-Diagrams reached an accuracy rate of 83% pre-training.

Following the acquisition of clinical characteristics of OLP, the diagnostic performance of each platform demonstrated significant improvement. Taking Claude Opus as an example, its sensitivity in diagnosing buccal mucosa lesions improved from 16% prior to training to 56%. Although the improvements in Chat-4O and Chat-Diagram were not as pronounced as those of Claude, their overall accuracy rates exceeded that of Claude Opus and demonstrated statistical significance (*p* < 0.05). The substantial improvement data suggest that Claude excels in deep learning and image feature extraction; however, considerable potential for enhancing overall recognition effectiveness remains.

## 4. Discussion

The rational use of AI platforms can significantly enhance both the diagnostic efficiency and accuracy for OLP. We designed a prospective, multicenter study protocol to systematically evaluate the practical application of mainstream AI platforms in diagnosing OLP. Notably, following targeted learning, the recognition capabilities of AI can match those of specialized mucosal disease clinicians and, in some instances, even exceed their performance [25]. Our horizontal comparison of various AI platforms revealed that the diagnostic performance of current mainstream AI systems for OLP is neither consistent nor stable [26]. Among these, the Chat-Diagrams platform demonstrated superior recognition performance compared to both ChatGPT-4O and Claude Opus across most affected sites. This finding underscores the necessity for caution in selecting appropriate AI platforms and providing precise instructions in clinical applications to avoid over-reliance on AI-generated diagnoses, thereby ensuring the reliability of final clinical decisions made by healthcare providers [27].

The application of AI technology in disease image recognition reveals immense potential. In recent years, various research teams have investigated the performance of AI in analyzing images from disease-related CT scans [28], chest X-rays, and diverse skin conditions [29]. The processes for recognizing these images on AI platforms exhibit commonalities, as they aggregate and summarize disease features through neural network learning; however, the effectiveness of AI processing varies across different image types. Currently, while AI has achieved commendable results in image recognition, its performance in various fields has not yet reached a level suitable for completely independent diagnosis. Nevertheless, it provides significant convenience to clinical practitioners. In the recognition study of OLP, the Chat-Diagrams platform demonstrated overall performance comparable to that of specialized clinicians [30], highlighting its considerable potential.

Notably, even within the same AI platform, diagnostic performance can vary significantly based on the lesion’s location. The diagnostic accuracy for the buccal mucosa, which has a high incidence rate, is relatively high, followed by the tongue, while performance for the gingiva and other less common sites tends to be lower. This variation may be attributed to the number of cases collected from each site and their respective incidence rates. In the context of OLP, the buccal mucosa has the highest incidence, resulting in a larger pool of clinical samples that provide AI platforms with more learning opportunities; a greater sample size enhances the comprehensive learning of imaging features. Furthermore, lesions in areas such as the gingiva exhibit greater diversity and complexity compared to those in the buccal mucosa, increasing the likelihood of confusion with other mucosal diseases and complicating feature extraction and classification. To enhance the intelligent diagnostic capabilities of AI platforms in the future, it will be crucial to utilize large-scale datasets with manual annotations, with a focus on improving AI recognition and classification abilities for rare disease sites and atypical cases.

Targeted training is a crucial method for enhancing the independent thinking capabilities of AI platforms in clinical environments. By focusing training on a specific disease, substantial improvements in diagnostic accuracy and efficiency can be achieved. This targeted learning not only positions AI as a valuable support tool for clinicians but also highlights the importance of specialized training in clinical practice. Our further analysis reveals that the performance of AI in image recognition is closely linked to the specific training it receives. Targeted feature learning for diseases can significantly enhance the AI recognition capabilities for relevant images. As illustrated in Table 2, following targeted training on OLP case images, the overall and site-specific recognition accuracy for the Chat-4O, Chat-Diagrams, and Claude platforms demonstrated substantial improvements. Notably, Claude Opus exhibited significant improvement in diagnostic capability pre-training; however, its overall performance remains inferior to that of other platforms. This phenomenon underscores the critical role that targeted medical knowledge learning plays in bolstering AI’s diagnostic capabilities. In the future development of intelligent diagnostic tools, it is essential to integrate all aspects of medical features, including clinical presentations and pathological results, to guide AI in creating the most suitable, comprehensive, and specialized diagnostic models.

The learning processes of clinicians and AI platforms shares fundamental similarities. Physicians enhance their diagnostic skills through continuous reflection and data aggregation while addressing patient issues. Similarly, AI platforms train their models using extensive datasets of images and information, updating recognition patterns and optimizing algorithms to improve their capabilities. For physicians, experience is built from patient histories, clinical manifestations, and pathological examinations, whereas AI relies on training with large datasets, employing deep learning techniques to automatically identify patterns and construct appropriate models [31,32].

This research process is subject to specific limitations. The limited number of clinical samples collected, particularly from relatively rare locations, has affected the reliability and stability of evaluating the AI platform’s recognition capabilities. Therefore, future studies should aim to expand the sample size to yield more robust results. Additionally, this study primarily focused on the recognition and diagnosis of OLP and did not include differential diagnoses of other oral mucosal diseases, which restricts the AI model’s discriminative ability for other conditions. Future development of AI models should incorporate a broader range of mucosal diseases to enhance the utility and accuracy of AI in complex clinical scenarios.

## 5. Conclusions

The considerable potential exhibited by AI platforms in the diagnostics of oral medicine establishes a robust foundation for AI-assisted systems. With the increasing accumulation of big data from oral disease images and advancements in deep learning algorithms, AI platforms are anticipated to significantly enhance diagnostic accuracy and efficiency in clinical practice. Nevertheless, in clinical practice, the most reliable method for diagnosing a disease remains pathological examination. While future AI platforms are expected to become powerful tools in assisting the diagnosis of various oral diseases, the gold standard for definitive diagnosis should still be pathological examination. We envision a future where AI becomes an indispensable tool in medicine, offering innovative diagnostic and treatment methodologies for both clinicians and patients [27,33].

## Figures and Tables

**Table 1:** Patient information.

Affected Areas of OLP		Buccal Mucosa	Tongue	Vestibular Sulcus	Gingiva	Cheilosis
Samples		70	35	7	10	6
Age (Years)		45.76 ± 13.02	46.63 ± 14.54	48.71 ± 11.24	50.00 ± 6.63	51.17 ± 5.23
Sex	Male	32	14	4	4	1
	Female	38	21	3	6	5

Note: Collection on the affected sites, age, and gender of OLP patients. Age: Mean ± SD.

**Table 2:** Accuracy of recognition across different anatomical sites for each platform.

Different Sites	Number of Cases	Non-Pre-Training			Pre-Training		
		Chat-4O	Chat-Diagrams	Claude	Chat-4O	Chat-Diagrams	Claude
Buccal mucosa	70	56 (80%)	60 (86%)	11 (16%)	66 (94%)	65 (93%)	39 (56%)
Tongue	35	11 (31%)	16 (46%)	3 (9%)	17 (49%)	19 (54%)	14 (40%)
Vestibular sulcus	7	4 (57%)	7 (100%)	3 (43%)	5 (71%)	7 (100%)	7 (100%)
Gingiva	10	3 (30%)	1 (10%)	1 (10%)	6 (60%)	6 (60%)	2 (20%)
Cheilosis	6	2 (33%)	3 (50%)	1 (17%)	5 (83%)	5 (83%)	2 (33%)
Overall	128	76 (59%)	87 (68%)	19 (15%)	99 (77%)	102 (80%)	64 (50%)

Note: Using the bootstrapping statistical method, the means and their 95% confidence intervals (CIs) were calculated for each group.

**Table 3:** Analysis of differences in diagnostic accuracy: Chat-4O, Chat-Diagram, Claude Opus.

	Chat-4O	Chat-Diagrams	Claude	*p*
Non-pre-training	76 (59%)	87 (68%)	19 (15%)	<0.01
Pre-training	99 (77%)	102 (80%)	64 (50%)	<0.01

Note: Inter-group comparison uses one-way ANOVA. A *p*-value < 0.05 is considered statistically significant.

## Data Availability

The datasets presented in this article are not readily available, due to privacy limitations.
